# An anti-PDGFRβ aptamer for selective delivery of small therapeutic peptide to cardiac cells

**DOI:** 10.1371/journal.pone.0193392

**Published:** 2018-03-07

**Authors:** Alessandra Romanelli, Alessandra Affinito, Concetta Avitabile, Silvia Catuogno, Paola Ceriotti, Margherita Iaboni, Jessica Modica, Geroloma Condorelli, Daniele Catalucci

**Affiliations:** 1 Department of Pharmacy, University of Naples “Federico II”, Naples, Italy; 2 Department of Molecular Medicine and Medical Biotechnology, University of Naples “Federico II”, Naples, Italy; 3 Institute of Biostructures and Bioimaging, National Research Council, Naples, Italy; 4 Institute of Experimental Endocrinology and Oncology "G. Salvatore "IEOS-CNR, Naples, Italy; 5 Humanitas Clinical and Research Center, Rozzano (Milan), Italy; 6 Institute of Genetics and Biomedical Research, Milan Unit, National Research Council, Milan, Italy; IRCCS-Policlinico San Donato, ITALY

## Abstract

Small therapeutic peptides represent a promising field for the treatment of pathologies such as cardiac diseases. However, the lack of proper target-selective carriers hampers their translation towards a potential clinical application. Aptamers are cell-specific carriers that bind with high affinity to their specific target. However, some limitations on their conjugation to small peptides and the functionality of the resulting aptamer-peptide chimera exist. Here, we generated a novel aptamer-peptide chimera through conjugation of the PDGFRβ-targeting Gint4.T aptamer to MP, a small mimetic peptide that via targeting of the Ca_v_β2 subunit of the L-type calcium channel (LTCC) can recover myocardial function in pathological heart conditions associated with defective LTCC function. The conjugation reaction was performed by click chemistry in the presence of N,N,N’,N’,N”-pentamethyldiethylenetriamine as a Cu (I) stabilizing agent in a DMSO-free aqueous buffer. When administered to cardiac cells, the Gint4.T-MP aptamer-peptide chimera was successfully internalized in cells, allowing the functional targeting of MP to LTCC. This approach represents the first example of the use of an internalizing aptamer for selective delivery of a small therapeutic peptide to cardiac cells.

## Introduction

Therapeutic peptides for clinical applications have recently obtained increasing interest, reaching over 60 FDA-approved products on the market and more than 150 mimetic peptides in clinical trials [1, 2]. This achievement has been facilitated by a range of novel peptide technologies, allowing for dedicated chemical design strategies to avoid aggregation, increase solubility, and extend the stability of peptides [3, 4]. On the other hand, cell membrane permeability and low cell-specific targeting are still challenging issues and despite some encouraging results obtained with a combined use of cell penetrating peptides, such as TAT and R7W [5], these issues weaken the safe and efficient clinical application of current therapeutic peptides.

Recently, we developed a cell-penetrating therapeutic peptide (R7W-MP), which is endowed with the ability to recover myocardial contractility in pathological heart conditions via restoration of L-type calcium channel (LTCC) density at the plasma membrane [6, 7]. LTCC is a multi-protein complex composed of a pore-forming Ca_v_α1.2 unit and accessory subunits, such as the cytoplasmic Ca_v_β2, which is the chaperone of the pore unit. In the heart, LTCC plays a critical role in regulating cardiac contractility [8]. In line with this, acquired and genetically determined LTCC dysfunctions have been causally associated with various conditions of human cardiovascular pathologies [9–12]. R7W-MP is composed of a R7W cell-penetrating peptide fused to an 11 acid peptide (MP), which is the active component of the therapeutic molecule. MP, which mimics an amino acidic stretch of the C-terminal tail of the Ca_v_β2 cytosolic chaperone, is designed to specifically target the Tail Interacting Domain (TID) within the Ca_v_β2 globular domain, facilitating the restoration of Ca_v_α1.2 protein density at the plasma membrane in heart conditions associated with altered LTCC levels and function [6]. Mechanistically, R7W-MP restores at the plasma membrane the reduced protein levels of dysregulated Ca_v_α1.2 by acting on both reverse (degradation) and forward (maturation) trafficking of the channel protein, thereby recovering LTCC density. However, despite the encouraging results obtained for the therapeutic treatment of LTCC-related cardiac conditions, such as diabetic cardiomyopathy [6, 7], the use of R7W-MP faces limitations due to the broad tissue targeting of the R7W moiety, which is not cell specific and may thereby lead to potential side effects at other locations where the Ca_v_β2 target is expressed. This limitation highlights the critical need for the identification of novel and more cell-specific targeting carriers.

Aptamers, which are short single stranded oligonucleotides of DNA, RNA, or modified RNA and DNA, are emerging as a very interesting class of molecules that can fold into complex tertiary structures and bind with high affinity to a specific target [13]. In particular, isolated from combinatorial libraries by a Systematic Evolution of Ligands by Exponential enrichment (SELEX) process [14, 15], aptamers can be designed for the recognition of any surface receptor undergoing receptor-mediated cell internalization, thereby serving as cell-selective carriers for a secondary reagent. Thus, by conjugation to specific aptamers, off-target effects of therapeutic peptides might be greatly reduced [16]. Moreover, compared to other targeting ligands and monoclonal antibodies, aptamers show many additional advantages, such as low toxicity, high specificity, and superior stability in biological fluids.

Here, we addressed the specific delivery of MP to cardiac cells and developed a novel approach based on direct conjugation of the therapeutic peptide to a cell internalizing aptamer as carrier. In particular, we designed a novel aptamer-peptide chimera and conjugated the therapeutic MP directly to Gint4.T, an aptamer specifically binding to the platelet-derived growth factor receptor-β, PDGFRβ [17], which is a receptor expressed in cardiomyocytes [18]. Results obtained with the cell-targeting Gint4.T-MP chimera provide the proof-of-concept for a significant step forward towards a safe and selective use of MP for the treatment of cardiac disorders associated with LTCC abnormalities [9, 10, 19]. Furthermore, the development of aptamer-peptide conjugates has a broader applicability for the selective delivery and intracellular penetration of therapeutic peptides in several diseases, including cancer [13].

## Material and methods

### Peptide synthesis

Peptides were obtained by solid phase synthesis using standard protocols [20]. Peptides were purified by HPLC on a Phenomenex Axia Jupiter 4 μm Proteo 90 Å (250x21.2) column using a gradient of acetonitrile (0.1% TFA) in water (0.1% TFA) from 5 to 50% in 20 minutes and characterized by LC-MS on a LC-MS Agilent Technologies 6230 ESI-TOF using a Phenomenex Jupiter 3 μm C18 (150x2.0 mm) column.

MP sequence: NorLeu-DQRPDREAPRS. Calculated mass (Da): 1479.3510; found: 740.8723 [M+2H]^2+^, 494.2513 [M+3H]^3+^.

Scramble sequence: NorLeu-DQPPSRRDERA. Calculated mass (Da): 1479.3510; found: 740.8723 [M+2H]^2+^, 494.2513 [M+3H]^3+^.

### Protocol for the conjugation of peptides to the aptamer Gint4.T

5 nmol of Gint4.T aptamer, equipped with a 3’-propargyl adenosine at the 3’ end in place of standard adenosine (MW: 10700 Da) were reacted with 7.5 nmol of MP (or scramble, scr) in 100 mM Tris HCl, pH 7.5 buffer containing CuSO_4_ • 5H_2_O (5 μmol), ascorbic acid (0.1 μmol), and pentamethyldiethylenetriamine (PMDETA) (0.01% v/v) in a total reaction volume of 500 μL. The reaction proceeded at room temperature under argon for 2 hours. The crude was stored at -20°C. The sample was purified by PAGE purification on a 12% acrylamide 7 M urea gel, using Gint4.T as control.

RT-PCR of the purified sample was carried out to demonstrate the presence of the aptamer in the conjugate. 500 ng of Gint4.T and Gint4.T-MP were retro-transcribed with Reverse Transcriptase M-MuLV (Roche Life Science) and amplified with FIREPol DNA Polymerase (Microtech) using specific primers: Gint4.T FW: 5’ TAATACGACTCACTATAGGGTGTCGTGGGGCA; Gint4.T RV: 5’ TGTCGAATTGCATTTACT.

Alkaline hydrolysis of the pure sample was also performed to demonstrate the presence of the peptide moiety in the conjugate. Briefly, the conjugate (4.4 μg in 18 μL H_2_O) was diluted with 12 μL Na_2_CO_3_ (4 mM), NaHCO_3_ (46 mM) buffer, pH 9.2 and incubated at 95°C for 90 minutes. At the end of the reaction the crude was analyzed by LC-MS as described earlier.

### Cell culture conditions and treatment

Cardiac muscle cells (HL-1) were cultured in Claycomb medium (Sigma) supplemented with 10% FBS (Sigma), 100 U/ml penicillin, 0.1 mg/ml streptomycin (Euroclone), 1% Ultraglutamine 1 (Lonza), and 0.1M Norepirephrine (Sigma) in a gelatin/fibronectin pre-coated flask. The treatment with peptides (R7W-MP or R7W-scr) or aptamers (Gint4.T, Gint4.T, or Gint4.T-scr) was performed in serum-free medium (Opti-MEM I reduced-serum medium, Thermo Fisher Scientific). After 24 h, cells were collected and analyzed.

### Calcium assay

The Fluo-4 Direct Calcium Assay was performed as described by the manufacturer (Thermo Fisher Scientific). Briefly, HL-1 cells, pretreated as described, were stimulated with Fluo-4 Direct calcium reagent (Thermo Fisher Scientific) and signals were detected one hour post treatment. 10 mM Bay K8644 (Sigma) was added to cells and signals were immediately detected using a Synergy 4 instrument (BioTek). Results were analyzed using Prism 6.0 software (GraphPad Software, CA).

### Western blot analyses

Protein expression was evaluated by Western blot analyses. Samples were homogenized in RIPA buffer (150 mM NaCl, 10 mM Tris, pH 7.2, 0.1% SDS, 1% Triton-X100, 5 mM EDTA, 100 μM Na_3_VO_4_, 10 mM NaF), and Protease inhibitor 1X (Thermo Fisher Scientific), loaded onto a 4–10% acrylamide gradient gel, separated by electrophoresis, and transferred to a nitrocellulose membrane (Millipore). Primary antibodies against the following proteins were used: Ca_v_α1.2 (Abcam), GAPDH (14C10) (Cell Signaling Technology). Goat anti-mouse-HRP and Goat anti-rabbit-HRP (Thermo Fisher Scientific) were used as secondary antibodies. ECL (Millipore) was used for protein detection using a Chemidoc MP Imaging System (Biorad). Image J software (National Institutes of Health) was used for densitometry analysis.

### Statistical analysis

Prism 6.0 software (GraphPad Software, CA) was used to assess normality of the data using the Kolmogorov-Smirnov (K-S) test and for statistical comparisons using Dunn’s test.

## Results and discussion

### Aptamer-peptide conjugation

**Fig 1:**
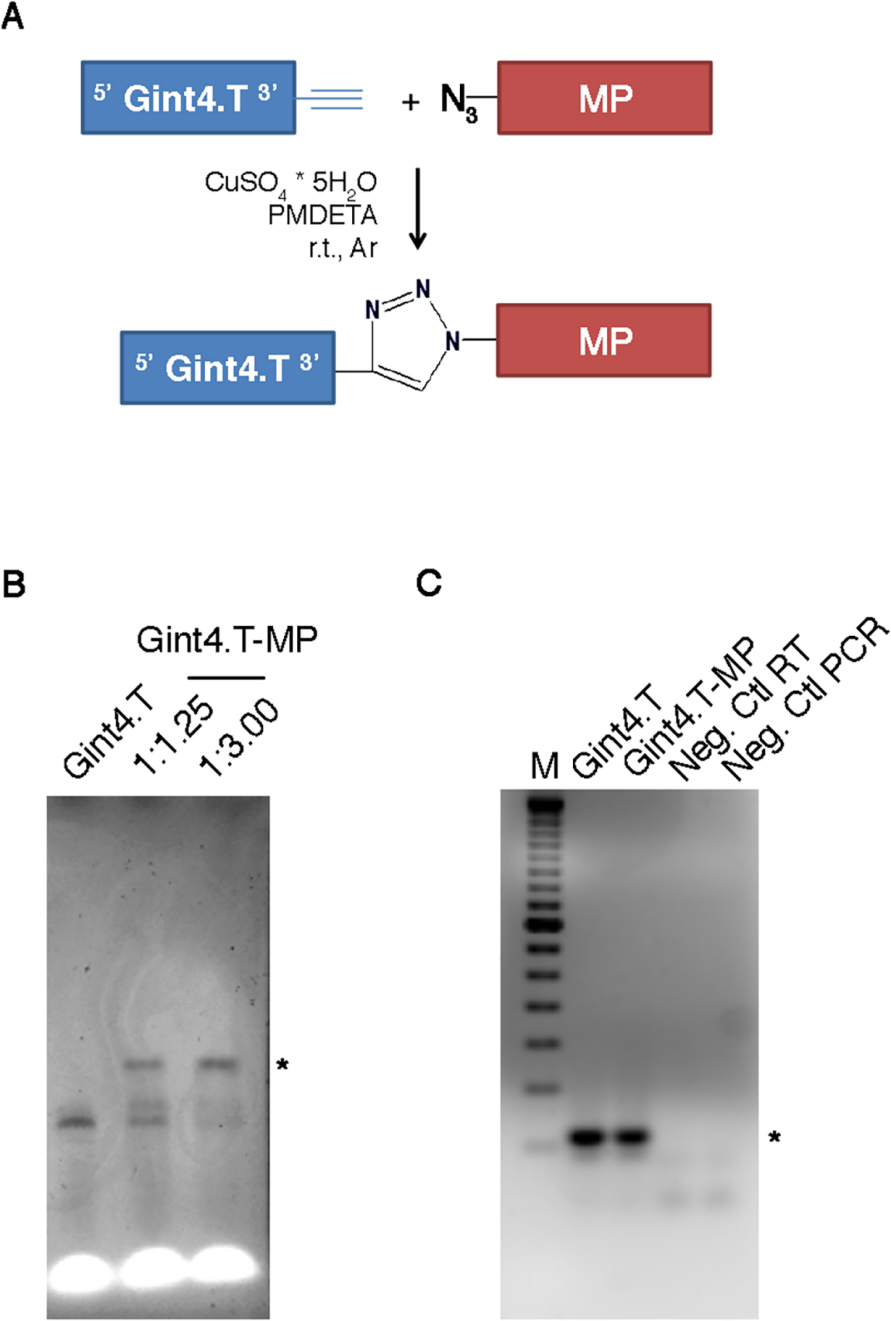
Gint4.T-MP conjugation. (A) Strategy for the conjugation of Gint4.T aptamer and MP peptide. (B) Analysis of click chemistry reactions between Gint4.T 3’-propargyl adenosine and MP by 12% acrylamide 7 M urea electrophoresis in two different reaction conditions: 1:1,25 (aptamer:peptide) ratio and 1:3 (aptamer:peptide) ratio. The star indicates the Gint4.T/peptide conjugates. (C) Characterization of the aptamer portion by RT-PCR. Gint4.T and Gint4.T-MP were reverse-transcribed, amplified, and loaded on a 3% agarose gel. The star indicates Gint4-T, which is 53 nt. Neg. Ctl RT and Neg. Ctl PCR refers to the mix of reverse-transcription and PCR without template.

To provide guidance for targeting of MP to the heart, we conjugated it to the Gint4.T aptamer, which we previously showed promotes the functional internalization of miRNAs and anti-miRs in a PDGFRβ-dependent manner [21, 22]. This was achieved through Cu (I)-mediated click chemistry, an approach poorly explored mainly due to difficulties associated with RNA degradation caused by Cu (I) disproportionation and subsequent redox reactions. Ligands, such as tris[(1-benzyl-1H-1,2,3-triazol-4-yl)methyl]amine (TBTA) [23] or the water-soluble tris(3-hydroxypropyl-triazolylmethyl)amine (THPTA) [24], have been employed to stabilize Cu (I) and prevent the metal disproportionation reaction. In addition, the combination of TBTA with DMSO has recently been employed for the successful conjugation of RNA to peptides [25]. Nonetheless, this possibility still faces critical issues as DMSO may affect RNA secondary structure [26] and thereby disturb the aptamer-mediated cell internalization. Thus, we here decided to perform all reactions in DMSO-free aqueous buffers using N,N,N’,N’,N”-pentamethyldiethylenetriamine (PMDETA) as a Cu (I) stabilizing agent for the conjugation of the RNA aptamer Gint4.T to MP. The advantage of PMDETA is its water solubility and ease to separate from the reaction mixture upon purification of the conjugate [27]. The sequence of the aptamer employed for the conjugation is identical to that previously reported by our group [17] with the exception of the 3’-propargyl adenosine, which was used for the conjugation in place of standard adenosine at the 3’ end. The introduction of 2’-fluoro modified pyrimidine bases grants an increased stability of the RNA aptamer. The reaction is performed between the aptamer and the MP peptide, which is modified at its N-terminus by an azido-norleucine residue (Fig 1A). The extent of the reaction, which smoothly proceeded in the buffer using a slight excess of the peptide in 2 hours, was monitored by migration analysis of the resulting high molecular weight product through urea-acrylamide electrophoresis (Fig 1B). Purification of the Gint4.T-MP conjugate was then achieved by PAGE and the final product assessed by RT-PCR analysis to confirm that the Gint4.T sequence is indeed present in the purified high molecular weight Gint4.T-MP (Fig 1C). To further characterize the conjugate and confirm the presence of the peptide in the isolated compound, purified Gint4.T-MP was subjected to alkaline degradation (to remove the RNA) followed by LC-MS analysis. Results showed a product with a molecular weight of 2135 Da consistent with the degradation of the aptamer up to the last two bases at the 3’ end (*i*.*e*. the clicked propargyl adenosine and the 2’-fluoro-cytosine, data not shown).

### Gint4.T-MP targets cardiac cells and restores LTCC protein levels

**Fig 2:**
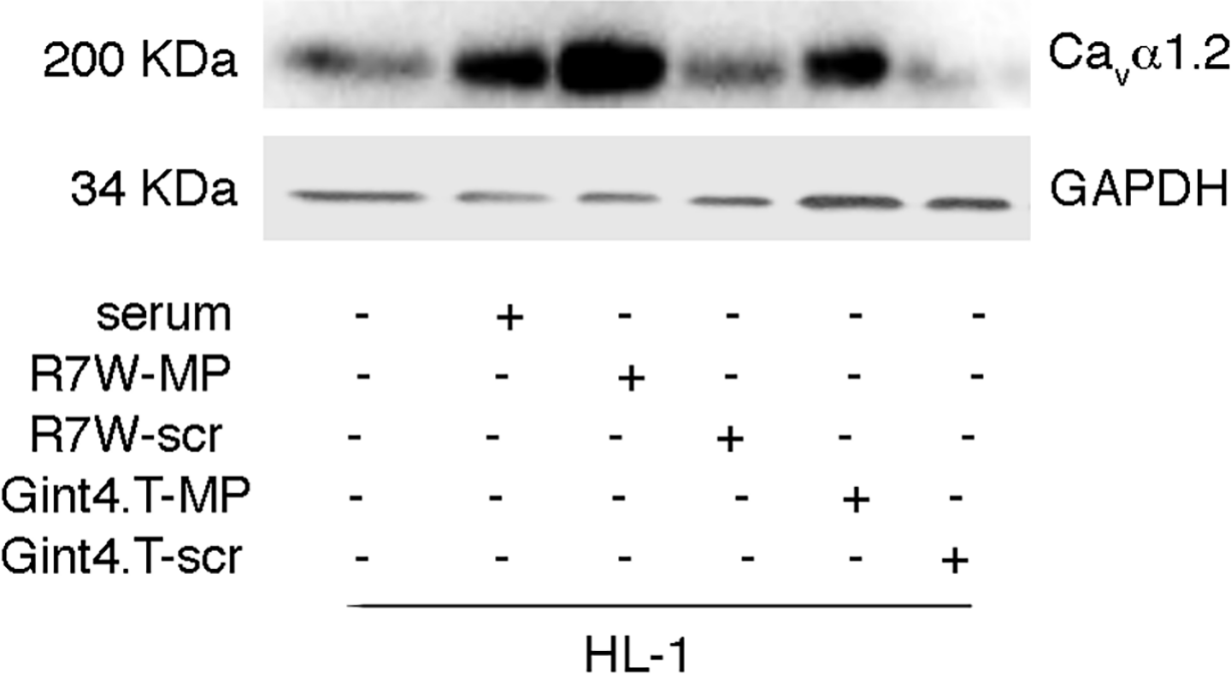
Gint4.T-MP conjugate reestablishes LTCC protein levels in HL-1 cardiac cells. Western Blot analysis for Ca_v_α1.2 in total protein lysates from HL-1 cells treated as indicated.

To determine whether the Gint4.T-MP chimera facilitates cell internalization of the MP, thus allowing its therapeutic effects on restoring Ca_v_α1.2 protein stability, we next explored its recovery effect in a cardiac context where all LTCC players are physiologically expressed. In line with this, HL-1 cardiac cells were subjected to serum starvation, which corresponds to a LTCC destabilizing condition [6], whereafter Ca_v_α1.2 protein levels were evaluated with or without treatment with either Gint4.T-MP or R7W-MP conjugates. As previously shown by our group [6], treatment of cardiac cells with R7W-MP, where the MP is fused to R7W cell penetrating peptide, allowed for a full restoration of Ca_v_α1.2 amounts, whereas no such effect was obtained with R7W fused to a scramble peptide (R7W-scr) (Fig 2). Notably, administration of Gint4.T-MP lead to similar effects and successfully recovered the protein levels of Ca_v_α1.2 to levels comparable to those obtained by R7W-MP administration or in serum-rich cells (Fig 2). On the other hand, no effect was obtained when the Gint4.T chimera conjugated to a scramble peptide (Gint4.T-scr) was applied.

### Gint4.T-MP recovers LTCC-dependent calcium fluxes in cardiac cells

**Fig 3:**
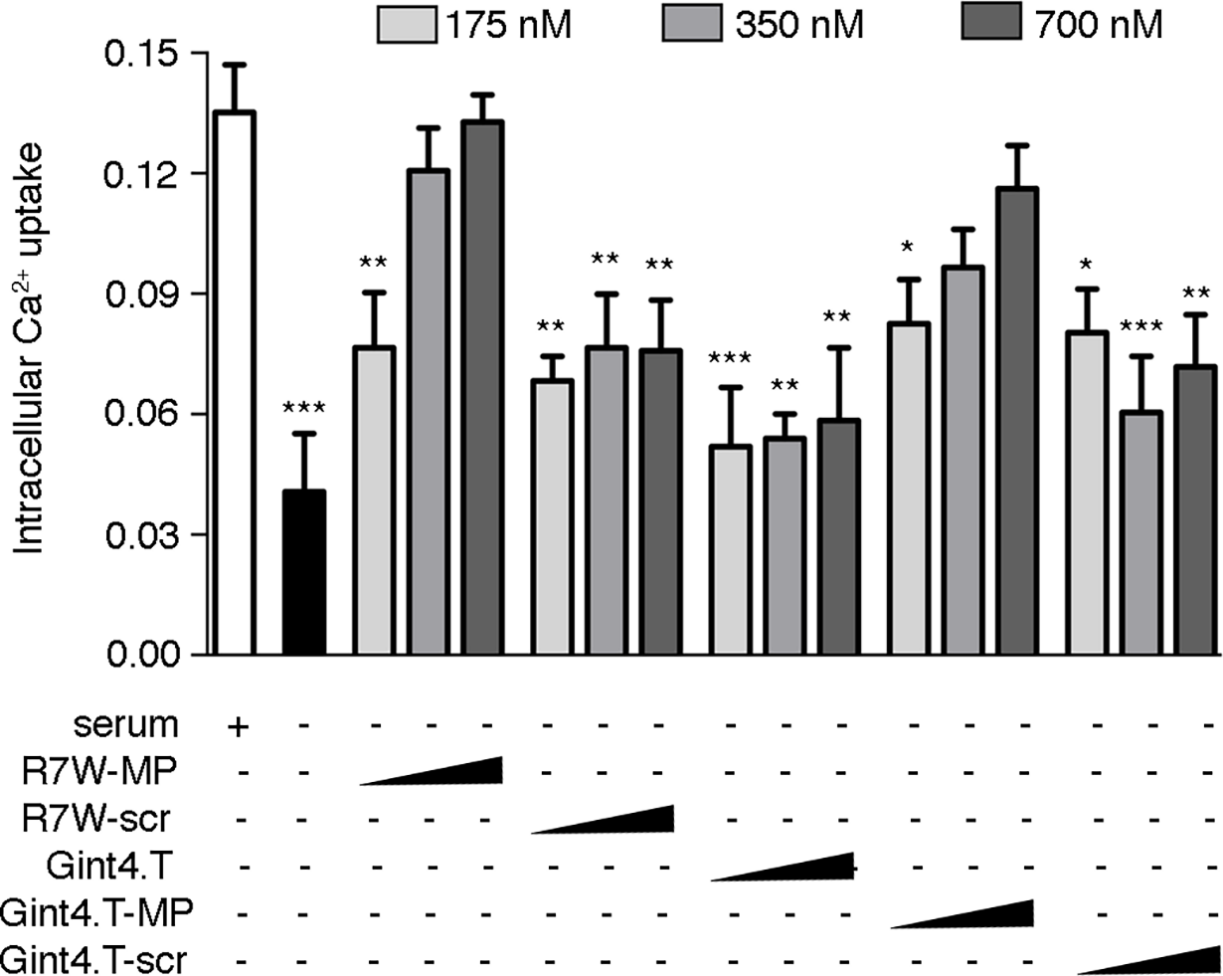
Gint4.T-MP conjugate reestablishes intracellular calcium levels in HL-1 cardiac cells. Intracellular calcium analysis in HL-1 cells treated as indicated. (n = 6, Dunnett’s multicomparison test). Individual data points are available upon request.

To functionally evaluate the Gint4.T-MP effect on channel density, we next performed a fluorescence-based assay of LTCC-dependent intracellular calcium fluxes in live cells. HL-1 cells were treated with the LTCC-specific agonist (BAYK8644), which through opening of the channel leads to an increase in intracellular calcium, which was measured in a fluorometric cell-based assay. As expected, a dramatic drop in intracellular calcium accumulation was observed in serum-starved HL-1 cardiac cells compared to the control state (Fig 3 and S1 Table). On the other hand, treatment with increasing doses of Gint4.T-MP resulted in an incremental recovery of LTCC-dependent intracellular calcium accumulation. This effect was not obtained when the same doses of either Gint4.T-scr or unconjugated Gint4.T were applied. The results achieved with Gint4.T-MP, which are causally linked to the effective intracellular targeting of MP to its cytosolic target Ca_v_β2, were similar to those obtained with the administration of the R7W-MP. Altogether, these data support the concept that the therapeutic MP, when conjugated to the cell internalizing Gint4.T aptamer, can be efficiently directed to PDGFRβ-expressing cells and internalized for functional targeting of LTCC.

Possessing key advantages over large proteins or antibodies, including easy synthesis and low toxicity, peptides are emerging as highly effective therapeutics for the treatment of important fatal diseases, such as neoplastic and cardiovascular diseases [1, 2]. However, although cell-penetrating therapeutic peptide chimeras with high therapeutic target specificity have been developed, including the R7W-MP peptide [6], the lack of selective targeting to the diseased organ and tissue is a severe obstacle for their effective translation to the clinic. Our demonstration that conjugation of R7W-free MP to an internalizing aptamer can mediate its uptake in cardiac cells and functional targeting of cytosolic Ca_v_β2, may open up for therapeutic applications that are more selective to the heart. Using Gint4.T, we provide the proof-of-concept that the approach is effective and might be exploited within the context of the growing number of therapeutic peptides. However, due to the binding of Gint4.T to the PDGFRβ cell-surface receptor, which in addition to cardiomyocytes is expressed also in other cell types, such as smooth muscle cells and fibroblasts [28–30], further studies are required to identify internalizing aptamers with higher selectivity for the heart.

Furthermore, our proposed conjugation is achieved under favorable conditions, which include *i*) the absence of co-solvents potentially affecting the structure of the aptamer, and *ii*) the use of a water-soluble and cost-effective Cu (I) stabilizing agent, such as PMDETA.

## Conclusions

In conclusion, we here report a strategy that provides more selective delivery of MP therapeutic peptide to cardiomyocytes. To the best of our knowledge, this represents the first example of the use of an internalizing aptamer for delivery of a small therapeutic peptide to cardiac cells.

## Supporting information

S1 Table Data from intracellular calcium analysis in HL-1 treated cells. (XLSX)

## Abbreviations

LTCC: L-type Calcium Channel
MP: mimetic peptide
PMDETA: N,N,N’,N’,N”-pentamethyldiethylenetriamine
scr: scramble
THPTA: tris(3-hydroxypropyl-triazolylmethyl)amine
TBTA: tris[(1-benzyl-1H-1,2,3-triazol-4-yl)methyl]amine
